# Hyperforin Inhibits Akt1 Kinase Activity and Promotes Caspase-Mediated Apoptosis Involving Bad and Noxa Activation in Human Myeloid Tumor Cells

**DOI:** 10.1371/journal.pone.0025963

**Published:** 2011-10-06

**Authors:** Faten Merhi, Ruoping Tang, Marion Piedfer, Julie Mathieu, Isabelle Bombarda, Murhaf Zaher, Jean-Pierre Kolb, Christian Billard, Brigitte Bauvois

**Affiliations:** 1 INSERM U872, Université Pierre et Marie Curie, Université Paris Descartes, Centre de Recherche des Cordeliers, Paris, France; 2 AP-HP, Département d'Hématologie, Hôpital St Antoine, Paris, France; 3 INSERM U685, Hôpital Saint-Louis, Paris, France; 4 ISM2-AD2M, UMR CNRS 6263, Université Paul Cézanne, Marseille, France; University of Kentucky College of Medicine, United States of America

## Abstract

**Background:**

The natural phloroglucinol hyperforin HF displays anti-inflammatory and anti-tumoral properties of potential pharmacological interest. Acute myeloid leukemia (AML) cells abnormally proliferate and escape apoptosis. Herein, the effects and mechanisms of purified HF on AML cell dysfunction were investigated in AML cell lines defining distinct AML subfamilies and primary AML cells cultured *ex vivo*.

**Methodology and Results:**

HF inhibited in a time- and concentration-dependent manner the growth of AML cell lines (U937, OCI-AML3, NB4, HL-60) by inducing apoptosis as evidenced by accumulation of sub-G1 population, phosphatidylserine externalization and DNA fragmentation. HF also induced apoptosis in primary AML blasts, whereas normal blood cells were not affected. The apoptotic process in U937 cells was accompanied by downregulation of anti-apoptotic Bcl-2, upregulation of pro-apoptotic Noxa, mitochondrial membrane depolarization, activation of procaspases and cleavage of the caspase substrate PARP-1. The general caspase inhibitor Z-VAD-fmk and the caspase-9- and -3-specific inhibitors, but not caspase-8 inhibitor, significantly attenuated apoptosis. HF-mediated apoptosis was associated with dephosphorylation of active Akt1 (at Ser^473^) and Akt1 substrate Bad (at Ser^136^) which activates Bad pro-apoptotic function. HF supppressed the kinase activity of Akt1, and combined treatment with the allosteric Akt1 inhibitor Akt-I-VIII significantly enhanced apoptosis of U937 cells.

**Significance:**

Our data provide new evidence that HF's pro-apoptotic effect in AML cells involved inhibition of Akt1 signaling, mitochondria and Bcl-2 members dysfunctions, and activation of procaspases -9/-3. Combined interruption of mitochondrial and Akt1 pathways by HF may have implications for AML treatment.

## Introduction

**Figure 1:**
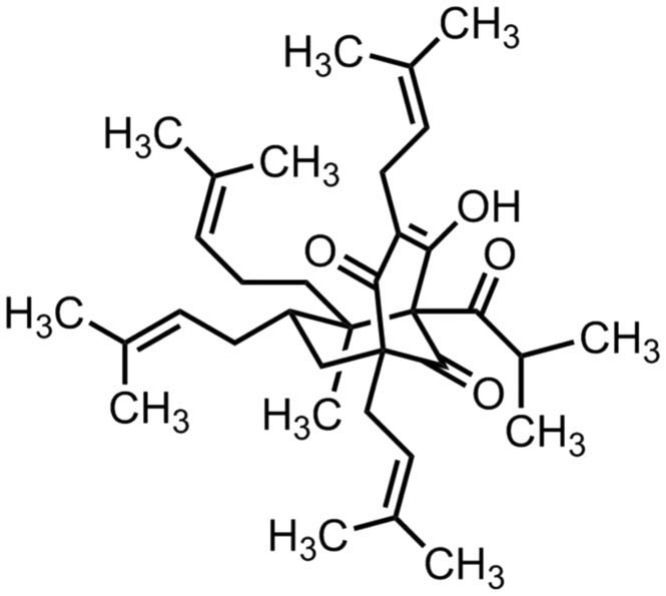
Chemical structure of hyperforin HF. 5-hydroxy-6R-methyl-1R,3,7S-tris(3-methyl-2-butenyl)-5S-(2-methyl-1-oxopropyl)-6R-(4-methyl-3-pentenyl)-bicyclo[3.3.1]non-3-ene-2,9 dione; C_35_H_52_O_4_; 536 g/mol. HF was purified according to the method described [75] (purity≥98%) and yielded less than 1% hypericin. HF was stored frozen in EtOH under conditions preventing its sensitivity to light, oxygen and aqueous solvents.

Compiled literature demonstrates that many natural products displaying anti-bacterial, anti-inflammatory or anti-tumoral effects represent potential interest for the development of new drugs especially designed for chemoprevention and chemotherapy of various diseases [1]–[3]. Extracts of the plant St John's wort, *Hypericum perforatum*, have been used for centuries in traditional medicine, notably for the treatment of depression [4]–[6]. Several biologically active compounds have been isolated and characterized from these extracts, including naphthodianthrones, flavonoids, and phloroglucinols such as hyperforin (HF) (Figure 1) [6]. HF has been identified as the major molecule responsible for the anti-depressant effects of this plant, and its neurobiological effects include neurotransmitter re-uptake inhibition, the ability to increase intracellular sodium and calcium levels, canonical transient receptor potential activation and N-methyl-D-aspartic acid receptor antagonism [4]–[6]. More recently, HF has been shown to have the ability to disassemble amyloid-beta aggregates *in vitro*, to decrease astrogliosis and microglia activation, as well as to improve spatial memory in a rat model, thus suggesting a potential role of HF in the treatment of Alzheimer's disease [7]. HF displays several other biological properties of potential pharmacological interest. They include antibacterial and antioxidant properties, and inhibitory effects on inflammatory mediators [5], [6]. HF acts as a dual inhibitor of 5-lipooxygenase and of cyclooxygenase 1, hence its interest in the treatment of inflammatory and allergic diseases connected to eicosanoids [5], [8]. HF inhibits the capacity of migration and invasion of different cell types, and inhibits urokinase, elastase and matrix metalloproteinases-2 and -9 involved in the degradation of the extracellular matrix, an early step in the process of angiogenesis [9]. The anti-angiogenic properties of HF were demonstrated *in vitro* and *in vivo* [10]. In cytokine-activated endothelial cells, HF prevents the activation of NF-κB, a transcription factor regulating numerous genes involved in cell growth, survival, angiogenesis and invasion [11]. As regards the immune system, HF inhibits the proliferation of peripheral blood mononuclear cells without displaying toxic effects whereas the incubation of endothelial cells with HF suppresses the proliferation of alloreactive T cells [5]. In the context of cancer, HF effectively inhibits the growth of a number of mammalian cancer cell lines *in vitro* [4], [5]. HF induces apoptosis in the K562 (chronic myeloid leukemia) and U937 (acute myeloid leukemia) cell lines through a caspase-dependent pathway [12], [13]. Our group previously reported that HF promotes *in vitro* a mitochondrial caspase-dependent apoptosis of primary malignant cells from patients with chronic lymphocytic leukemia [14] and this was associated with the activation of the pro-apoptotic protein Noxa [15].

Acute myeloid leukemia (AML) is a deadly disease characterized by the clonal expansion and accumulation of hematopoietic stem cells arrested at various stages of development. The latter are used to define distinct AML subfamilies [16]. Leukemia cells are unable to undergo (i) growth arrest, (ii) terminal differentiation, (iii) apoptosis in response to appropriate environmental stimuli, and disseminate from the bone marrow into peripheral tissues [16]. The conventional chemotherapeutic approach for AML patients is based on treatment combinating an anthracycline with cytarabine [16]. However AML therapy remains a challenge for clinicians because a large subset of patients are still refractory to primary therapies or relapse later. New drugs are currently in clinical development including inhibitors of tyrosine kinases, farnesyltransferase inhibitors, histone deacetylase inhibitors or deoxyadenosine analogues [16]–[18]. Other approaches are based on the identification of natural compounds capable of inducing apoptosis which is deficient in AML.

In this study, we sought to determine whether purified HF could show evidence of single drug activity in AML disease through inhibition of growth and survival processes. In addition, the underlying mechanisms and intracellular signaling pathways affected by HF in AML cells were investigated. Understanding HF's pro-apoptotic activity in AML may provide new therapeutic approaches for halting AML-associated survival.

## Results

### HF induces growth arrest and apoptosis in AML cell lines

**Figure 2:**
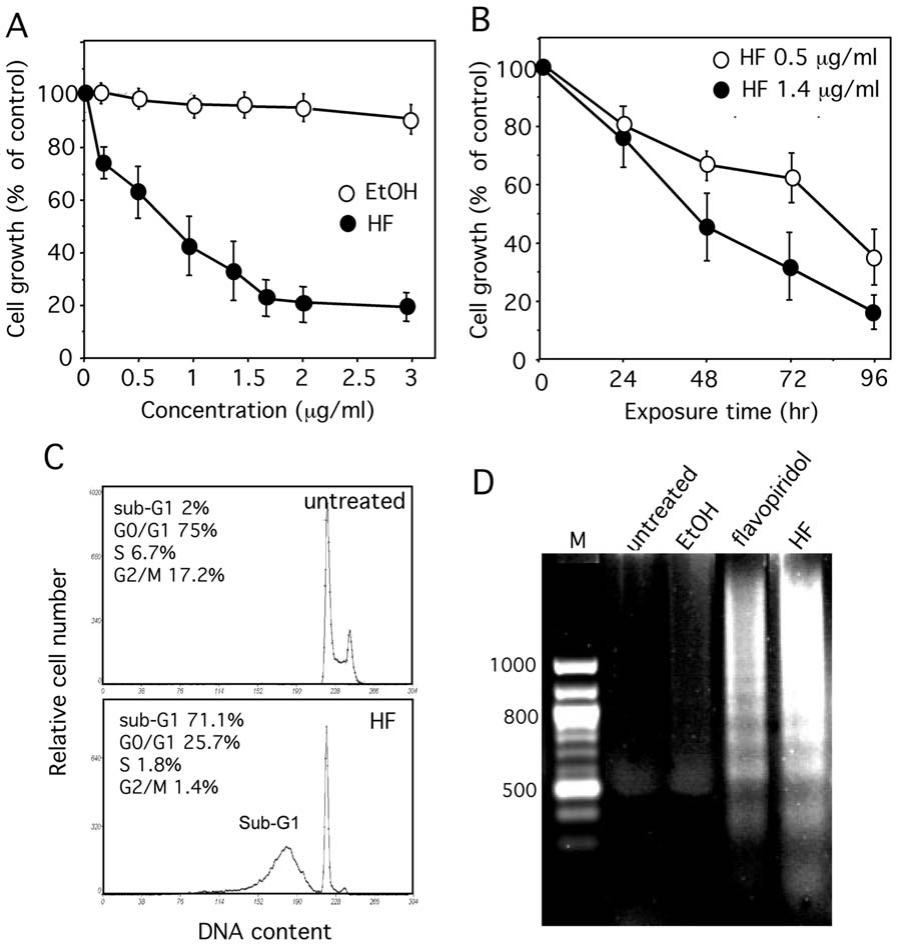
Effects of HF on U937 cell growth. U937 cells (10^5^/ml) were treated with HF (A) at the indicated concentrations for 72 h or (B) or with 0.5 and 1.4 µg/ml HF for the indicated times. Control EtOH (vehicle). Cell growth was measured by direct cell counting (in duplicates). Data are the mean ± SD of results from at least 6 independent experiments, each performed in duplicates. (C) U937 cells were incubated with 1.4 µg/ml HF for 72 h. Cells were stained with PI and DNA contents analyzed by flow cytometry. (D) DNA fragmentation in U937 cells treated for 72 h with 1.4 µg/ml HF, EtOH (vehicle) or 100 nM flavopiridol (F).

**Figure 3:**
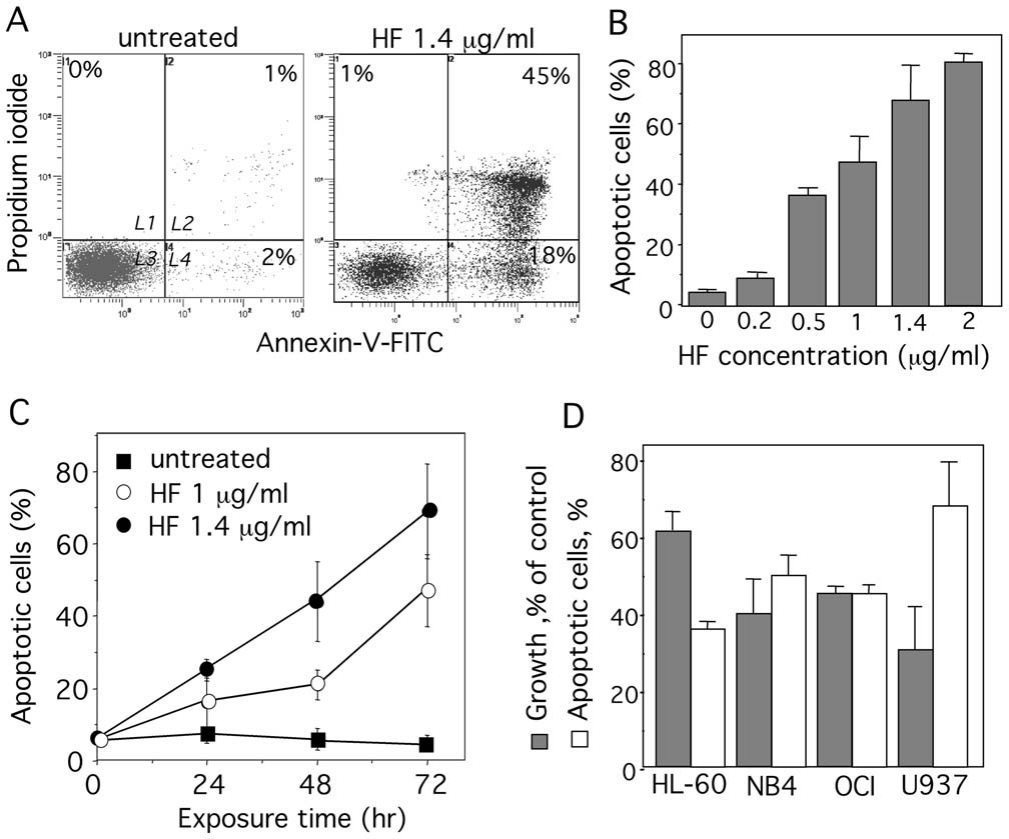
HF induces apoptosis in AML cell lines. (A) U937 cells were treated with 1.4 µg/ml HF for 72 h. Detection of apoptotic cells after annexin-V-FITC/propidium iodide staining and flow cytometry. Results are expressed as log PI fluorescence intensity (y-axis) vs log annexin-V-FITC fluorescence intensity (x-axis). L1, necrotic cells; L2, apoptotic + secondary necrotic cells; L3, healthy cells; L4, apoptotic cells. (B) Percent of apoptotic cells (L2+L4 gates) treated at the indicated concentrations for 72 h. Data are the mean ± SD of results from at least 4 independent experiments. (C) Percent of apoptotic cells (L2+L4 gates) treated with 1 or 1.4 µg/ml HF for the indicated times. Data are the mean ± SD of results from at least 4 independent experiments. (D) AML cell lines were treated 1.4 µg/ml HF for 72 h. Cell growth was quantified as % of untreated cells. Percent of apoptotic cells was determined as in (B). Data are the mean ± SD of results from at least 4 independent experiments (n = 4 for HL-60, OCI and NB4, n = 8 for U937). Basal apoptosis was ≤10% for HL-60 and U937, ≤20% for OCI and NB4).

We first examined the effects of HF on the growth and viability of U937 cells (monoblastic phenotype M5). Cells were cultured for 72 h in the absence or presence of increasing concentrations (0.2–3 µg/ml) of HF. Cell growth was markedly reduced in HF-treated samples, when compared with vehicle or no treatment (Figure 2A). The IC_50_ value (half-maximal inhibitory concentration) was around 1 µg/ml (1.8 µM). Kinetic studies revealed a time-dependent inhibitory effect of HF on U937 cell growth (Figure 2B). Cell growth inhibition was accompanied by reduction in DNA content to sub-G1 levels (Figure 2C) and internucleosomal DNA fragmentation (Figure 2D) characteristic of apoptosis. The positive control flavopiridol induced similar DNA fragmentation [19] (Figure 2D). Apoptosis was further confirmed by phosphatidylserine exposure at the cell surface, with consequential annexin-V-FITC binding whereas necrotic cells were detected by PI staining. Indeed, annexin-V binding was higher in HF-treated cells than in untreated cells (Figure 3A). The HF pro-apoptotic effects was dose- (Figure 3B) and time-dependent (Figure 3C). The other AML cell lines HL-60 (myeloblastic phenotype M2), NB4 (promyelocytic phenotype M3) and OCI-AML3 (myelomonocytic phenotype M4) were also found sensitive to the inhibitory effects of HF (Figure 3D).

### HF induces apoptosis in primary AML cells

**Figure 4:**
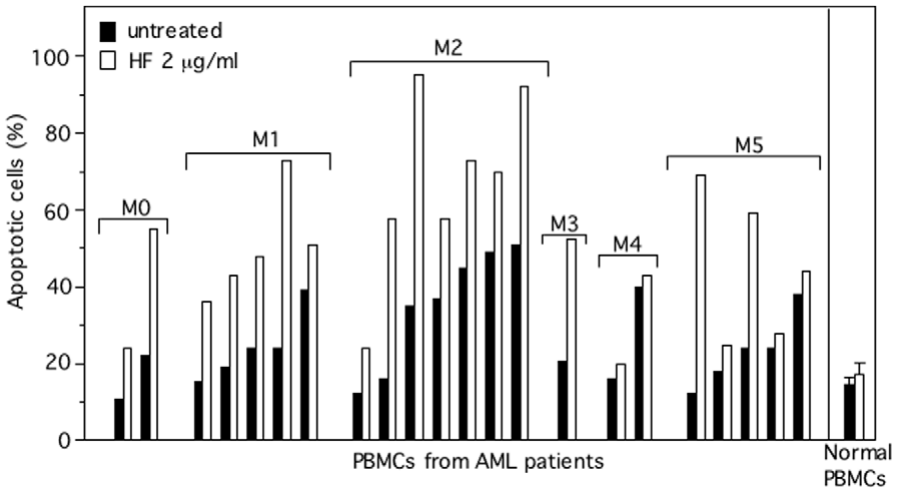
HF treatment induces apoptosis in primary AML cells. AML cells are characterized by the French American British (FAB) M0/undifferentiated, M1/myeloblastic, M2/myeloblastic with maturation, M3/promyelocytic, M4/myelomonocytic and M5/monoblastic phenotypes. PBMCs (10^6^/ml) from normal donors (n = 4) and AML patients were cultured with or without HF 2 µg/ml for 72 h, and then stained with annexin-V-FITC/PI and analyzed by flow cytometry to assess the percentage of apoptotic cells (L2+L4 gates) in untreated (basal apoptosis) and HF-treated cultures.

We investigated the effect of HF on peripheral blood mononuclear cells PBMCs obtained from 22 AML patients. The leukemic cells were exposed to HF (1, 1.4 and 2 µg/ml) and annexin-V binding was measured by flow cytometry after 24, 48 and 72 h. Cultured AML cells exhibited variable baseline levels of spontaneous apoptosis independently of the FAB subtype (Figure 4 for 72 h culture). As exemplified in Figure 4, the optimal effects of HF in inducing apoptosis of responding AML cells in culture were achieved by 72 h with 2 µg/ml HF. Exposure to HF increased apoptosis (≥20% of response) in 14 of the 22 samples from FAB-M0, M1, M2, M3 and M5 patients (Figure 4). The two samples from M4 patients did not respond to HF treatment. As previously reported [14], [15], HF did not markedly affect the viability of normal PBMCs (Figure 4).

### HF-induced apoptosis involves a caspase-dependent mechanism

**Figure 5:**
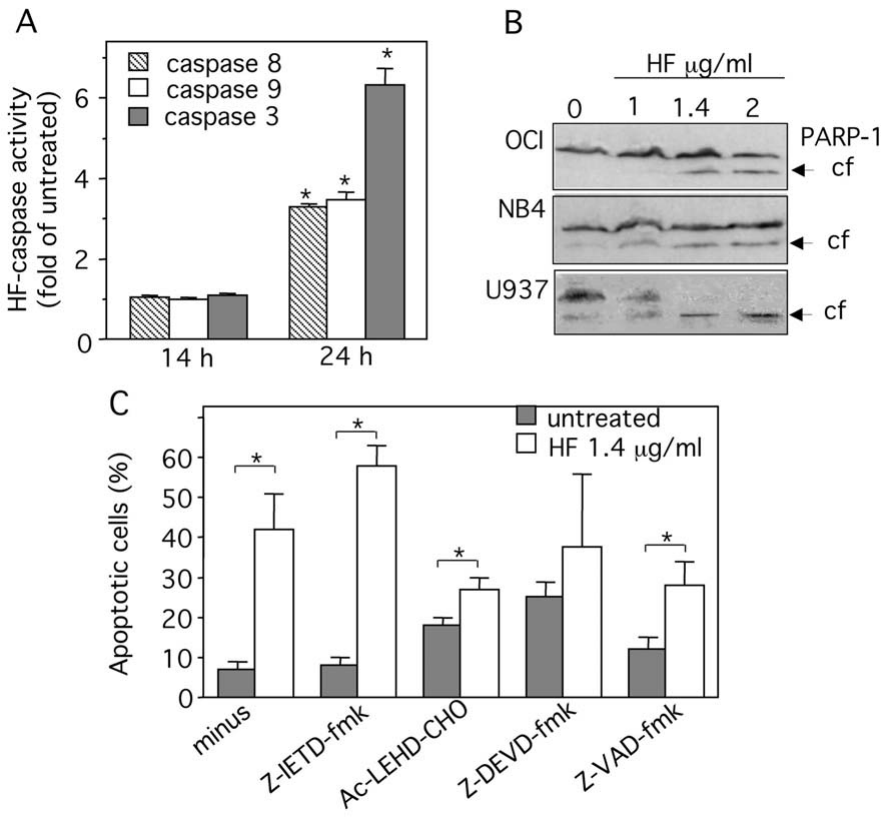
HF-induced apoptosis in U937 cells is caspase-dependent. (A) U937 cells were treated for 14 and 24 h with 1.4 µg/ml HF or left untreated. Caspase-8, -9 and -3 activities were determined using IETD-pNA (caspase-8), LEHD-pNA (caspase-9) DEVD-pNA (caspase-3) respectively. The release of pNA was measured at 405 nm. The data are expressed as fold-increase relative to respective untreated samples (basal values for caspase-8, -9 and -3 activities were respectively 23±0.02, 9±0.01 and 13±0.02 pmol pNA/60 min/µg protein). Data are from three determinations ± SD. (B) Lysates from U937, NB4 and OCI cells treated for 24 h with HF (1, 1.4, 2 µg/ml) were examined for PARP-1 expression by immunoblot. The arrows indicate the cleaved form (cf) of PARP-1. (C) U937 cells were incubated with 1.4 µg/ml HF for 48 h after 1 h-pretreatment with 50 µM Z-VAD-fmk (broad spectrum caspase inhibitor), Z-DEVD-fmk (caspase-3 inhibitor), Z-IETD-fmk (caspase-8 inhibitor) or Ac-LEHD-CHO (caspase-9 inhibitor). The percentage of apoptotic cells was determined after annexin-V-FITC/PI staining and flow cytometry (L2+L4 gates). Data are the mean ± SD of results from 3 independent experiments. *P<0.05 compared with untreated cells or cells pretreated with caspase inhibitors.

Caspases -3, -8 and -9 are important mediators of apoptosis; caspases -8 and -9 are the initiator caspases and caspase-3 is the “executioner enzyme” [20]. HF did not appear to activate caspases directly (B Bauvois, unpublished results). We studied the ability of U937 cell lysates to cleave chromogenic substrates of caspase-3, caspase-8 and caspase-9. Untreated U937 cells display similarly low baseline levels of all three caspase activities. The broad-spectrum caspase inhibitor Z-VAD-fmk inhibited caspase activities (data not shown). No changes were observed in the levels of caspase activities up to 14 h in HF-treated cells relative to the untreated cells (Figure 5A). By 24 h, cell treatment with HF resulted in a marked increase in all three caspase activities relative to untreated cells (Figure 5A). Accordingly, Western blot analyses showed that exposure of U937 cells to increasing concentrations of HF induced the cleavage of the downstream caspase substrate poly ADP-ribose polymerase-1 (PARP-1) (Figure 5B). Similar patterns of PARP-1 degradation were observed with NB4 and OCI-AML3 cells (Figure 5B).

To determine whether the activated caspases are involved in the apoptotic action of HF, we examined the effects of the broad-spectrum caspase inhibitor Z-VAD-fmk and selective inhibitors of caspase-3 (Z-DEVD-fmk), caspase-8 (Z-IETD-fmk) and caspase-9 (Ac-LEHD-CHO) on cell apoptosis as determined by annexin-V-FITC binding. HF-mediated apoptosis was blocked markedly by 50 µM Z-VAD-fmk, Ac-LEHD-CHO and Z-DEVD-fmk (Figure 5C), but not by Z-IETD-fmk (Figure 5C). In etoposide-treated U937 cells used as a positive control [21], this dose of Z-IETD-fmk was however efficient in blocking apoptosis (data not shown). Together these results show that HF induces apoptosis at least through the activation of caspases-9 and -3 in the intrinsic pathway.

### HF induces mitochondrial membrane depolarization, Bcl-2 downregulation and Noxa upregulation

**Figure 6:**
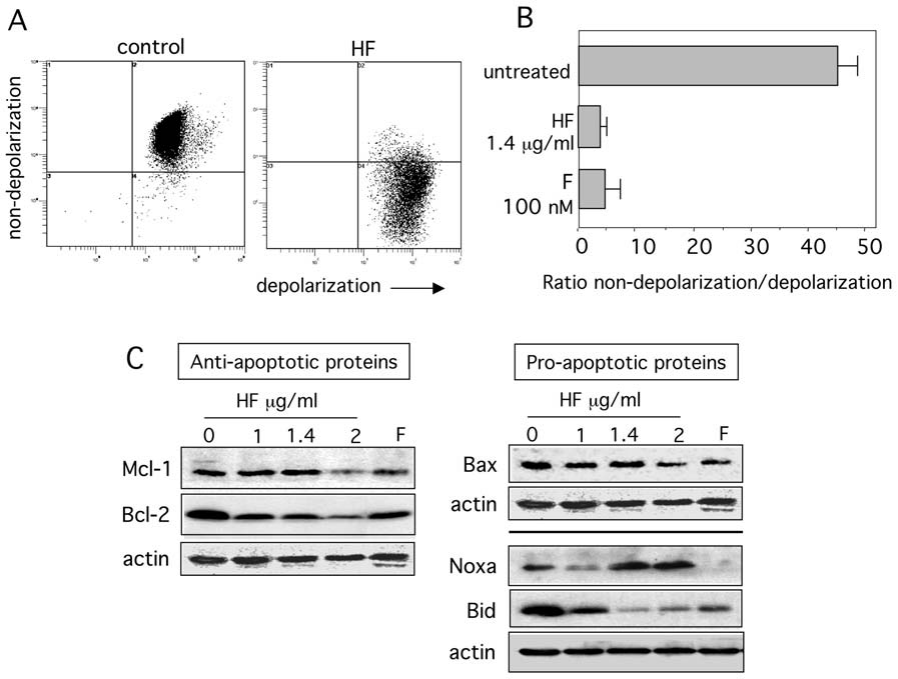
HF triggers a dissipation of the mitochondrial transmembrane potential associated with downregulation of Bcl-2 and Bid, and upregulation of Noxa. (A&B) U937 cells were cultured for 24 h in the absence or presence of 1.4 µg/ml HF or 100 nM flavopiridol (F) (a positive control of apoptosis in U937 cells). Then, cells were incubated for 15 min at 37°C with the fluorescent probe JC-1, subsequently washed and distributed in triplicates in the wells of a microtitration plate. Green and red fluorescences were measured. The loss of mitochondrial membrane potential is characterized by a a significant shift of the red (polarization) fluorescence to the green (depolarization) fluorescence. A) Representative histograms showing flow cytometry analysis of polarization and depolarization. B) Quantification of the polarization to the depolarization ratio. Data are expressed as mean ± SD of three determinations. (C) U937 cells were treated with 1, 1.4 or 2 µg/ml HF or 100 nM F for 24 h, after which lysates were subjected to Western blots using antibodies recognizing Bax, Bid, Bcl-2, Mcl-1, Noxa and actin. Two experiments representative of four are shown.

To confirm the intrinsic pathway's involvement in HF-induced apoptosis, we investigated the role of the mitochondria. In a fluorescence-based assay, the exposure of U937 cells to HF (1.4 µg/ml) and flavopiridol (positive control, 100 nM) induced a marked decrease in the mitochondrial membrane potential (Figure 6A&B). Mitochondrial membrane depolarization can result from the action of pro-apoptotic and/or anti-apoptotic members of the Bcl-2 family [20]. We therefore measured expression levels of Bcl-2 and Mcl-1 anti-apoptotic proteins and Bax and Noxa pro-apoptotic proteins before and after HF treatment. Untreated U937 cells expressed high levels of Bcl-2, Mcl-1, Bax and Noxa (Figure 6C). At 24 h culture, HF downregulated Bcl-2 in a dose-dependent manner while the levels of Mcl-1 appeared to be diminished for 2 µg/ml HF (Figure 6C). Moreover, HF upregulated Noxa in a dose-dependent manner whereas no significant changes were observed in the levels of Bax at 1 and 1.4 µg/ml HF when normalized to actin levels (Figure 6C). Bax levels were slightly diminished for 2 µg/ml HF (Figure 6C). Consistently with previous studies in U937 cells [22], [23], Bcl-2, Bax and Mcl-1 levels were lower in flavopiridol-treated cells than in untreated cells (Figure 6C). Bid is a known substrate of caspases, and the production of truncated Bid (tBid) can activate the intrinsic pathway of apoptosis [24]. Untreated cells expressed high levels of Bid, and HF treatment led to a concentration-dependent loss of intact Bid (Figure 6C). We were however unable to detect the formation of tBid fragments (14–15 kDa) (data not shown).

### HF suppresses Akt1 kinase activity and phosphorylations of Akt1 and Bad

**Figure 7:**
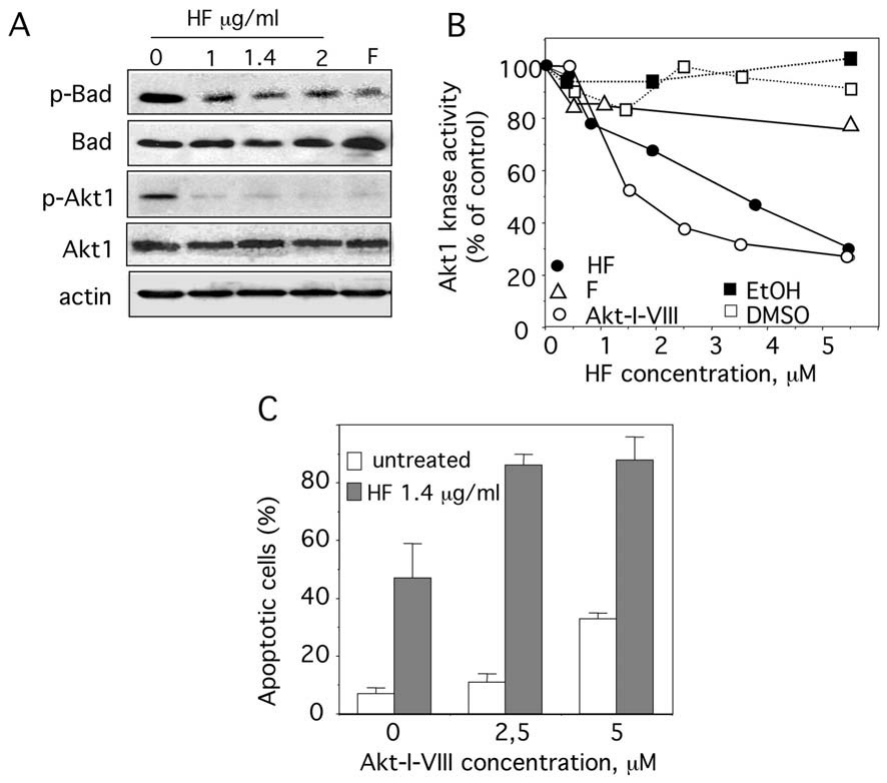
HF induces the dephosphorylation of Bad and Akt, and inhibits Akt1 kinase activity. (A) U937 cells were treated with 1, 1.4 or 2 µg/ml HF or 100 nM F for 24 h, after which lysates were subjected to Western blots using antibodies recognizing Ser^136^-p-Bad, Bad, Ser^473^-p-Akt1, Akt1 and actin. One experiment representative of three is shown. (B) Akt1 kinase activity was determined using a kinase assay kit obtained from Enzolifesciences France using recombinant active Akt1 in the absence or presence of increasing concentrations (0.5–5.5 µM) of Akt1/2 inhibitor Akt-I-VIII, F (flavopiridol), HF or vehicles EtOH (for HF) and DMSO (for F and Akt-I-VIII). Of note, 2.5 µM of HF corresponds to 1.4 µg/ml. Percent of Akt kinase activity was determined. (C) The percentage of apoptotic cells was determined after annexin-V-FITC/PI staining and cytometry, under control conditions, or 1.4 µg/ml HF or Akt-I-VIII (2.5, 5 and 10 µM) and in combination treatment for 48 h. Data are the mean ± SD of results from 2 independent experiments.

Bad, a member of Bcl-2 family is a direct downstream target of Akt1 [25]. Dephosphorylation of Bad on Ser^136^ induces apoptosis in primary AML cells [26]. U937 cells were negative for Ser^112^-phospho(p)-Bad (data not shown) and positive for Ser^136^-p-Bad (Figure 7A). A marked decrease in the level of Ser^136^-p-Bad was seen in response to HF while there was no change in the levels of total Bad (Figure 7A). Since active Ser^473^-p-Akt1 can negatively regulate apoptosis through phosphorylation of Bad on Ser^136^ [27], we measured the level of Ser^473^-p-Akt1 in U937 cells before and after HF treatment. Accordingly, the amount of Ser^473^-p-Akt1 was decreased in HF-treated U937 cells at 24 h while the amount of total Akt1 protein was not affected (Figure 7A). Consistently with previous studies in myeloid cells [28], [29], the levels of Ser^473^-p-Akt1 were also decreased in flavopiridol-treated cells (Figure 7A). We thus examined whether HF might directly inhibit the kinase activity of Akt1 *in vitro* using an ELISA that utilizes a specific synthetic peptide as a substrate for active recombinant Akt1 and a polyclonal antibody that recognizes the phosphorylated form of the substrate. Flavopiridol and the highly specific allosteric Akt1/2 inhibitor Akt-I-VIII were also assayed for their ability to inhibit Akt1 activity. As expected, Akt-I-VIII suppressed Akt1 kinase activity with a IC_50_ value of 1.3 µM (Figure 7B). Surprisingly, HF showed also a marked inhibitory activity for Akt1 with a IC_50_ value of 2.5 µM (1.4 µg/ml) (Figure 7B). In contrast, flavopiridol did not markedly affect Akt1 activity (Figure 7B). As demonstrated in Figure 7C, the inhibition of Akt1 with Akt-I-VIII induced a dose-dependent apoptosis in U937 cells. Furthermore, combined treatment with Akt-I-VIII and HF significantly increased the percentage of apoptotic cells as compared to that of HF or Akt-I-VIII alone (Figure 7C). These data strongly suggest that the pro-apoptotic effect of HF is at least in part mediated by inhibition of the Akt pathway, with suppression of Akt1 kinase activity and downregulation of the phosphorylated form of Bad.

### NF-κB pathway is not involved in HF-mediated cell growth arrest and apoptosis

**Figure 8:**
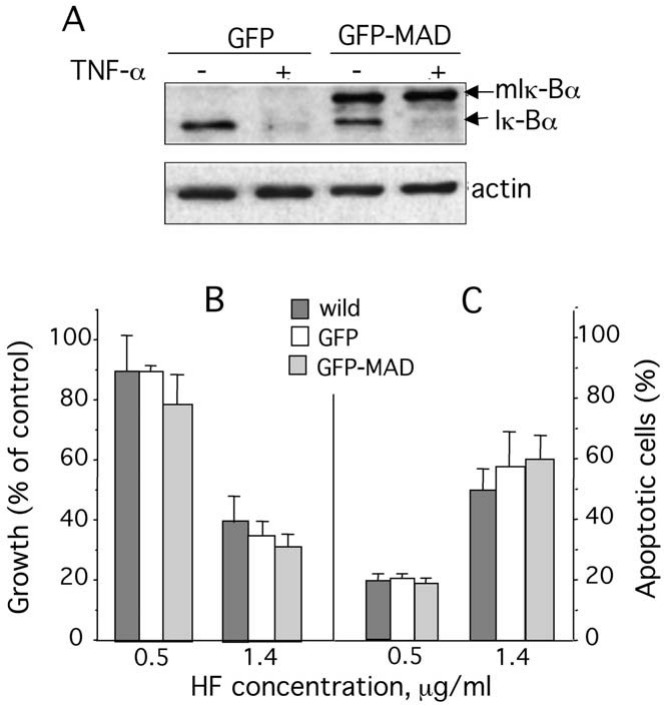
HF-induced apoptosis is independent on NF-κB activation. (A) NB4/GFP cells and NB4/GFP-MAD cells were left untreated or were incubated for 15 min with TNF-α (20 ng/ml), after which lysates were subjected to Western blots using antibodies recognizing Iκ-Bα and actin. (B) cell growth and (C) apoptosis of wild NB4, NB4/GFP and NB4/GFP-MAD cells (10^5^/ml) treated with HF at the indicated concentrations for 72 h. Data are the mean ± SD of results from 4 independent experiments.

Studies have linked AML cell proliferation and survival to NF-κB signaling pathway [30]. To assess whether NF-κB signaling was involved in the inhibition of AML cell growth by HF, we used stable transformant NB4 cells expressing a repressor form of Iκ-Bα, Iκ-Bα (A32/36) which acts like a constitutive repressor of NF-κB activation [31]. The wild-type Iκ-Bα was present in NB4/GFP and NB4/GFP-MAD cells and, as expected, was degraded following treatment with TNF-α (Figure 8A). A band corresponding to mutated Iκ-Bα was seen in the NB4/GFP-MAD cells only, but was not affected by TNF-αtreatment (Figure 8A). As reported [31], the expression of Iκ-Bα (A32/36) had no effect on the cell viability and the proliferation rate of NB4-GFP-MAD cells compared to parental or NB4-GFP cells (data not shown). In the presence of increasing concentrations of HF (0.5 and 1.4 µg/ml), the growth of NB4-GFP-MAD cells for 72 h was inhibited to the same rate than parental NB4 and NB4-GFP cells (Figure 8B). In parallel, the levels of cell death were similar in parental, GFP and GFP-MAD cells (Figure 8C). These data indicate that HF-mediated apoptosis did not implicate the NF-κB pathway.

## Discussion

HF exhibits anti-proliferative and pro-apoptotic activities towards various cancers cells [4]. Akt1 signaling plays a major role in AML survival and proliferation. The outcome of the present study indicated that HF promoted apoptosis of established AML cell lines defining distinct AML phenotypes and primary AML cells cultured *ex vivo*. Our major findings showed that HF blocked Akt1 activation by inhibiting its phosphorylation and kinase activity; suppressed Bad phosphorylation which can activate its pro-apoptotic function; activated the intrinsic pathway of cell death by targeting mitochondria, anti-apoptotic Bcl-2, pro-apoptotic Noxa, and caspases −9 and −3 in AML U937 cells.

Upon addition of HF in culture, the AML U937 cell line (FAB M5/monoblast) underwent growth arrest followed by apoptosis, as evidenced by accumulation of cells in the sub-G1 phase, phosphatidylserine externalization and DNA fragmentation. HF also exhibited antiproliferative and pro-apoptotic properties in AML cell lines characterized by distinct FAB subtypes, HL-60 (M2/myeloblast), NB4 (M3/promyelocyte) and OCI (M4/myelomonocyte). All these cell lines have the abilities to differentiate into monocytes/macrophages while NB4 and HL-60 cells can differentiate into granulocytes. Analysis of morphologic and phenotypic features specific to differentiating myeloid cells toward granulocytes or monocytes/macrophages showed that differentiation was not achieved in HF-treated cell lines (F Merhi, unpublished results). This indicates that HF-induced cell death was not a consequence of full terminal maturation. The results in AML cell lines were confirmed in primary AML cells with HF-mediated apoptosis observed in 14 of the 22 AML samples tested. Unlike samples with FAB M0, M1, M2, M3, M5, the 2 AML samples with FAB M4 did not respond to HF treatment. The impact of HF on a larger number of AML samples from patients with the M4 subtype needs to be investigated**.** Besides the direct anti-tumor effect of HF on malignant cells, attention will have to be laid on T cells and their antitumor responses from AML samples, before and after *in vitro* HF treatment. Percentages of residual T cells in AML samples will be evaluated and T cell responses analyzed. Such a potential to activate antitumor directed immune responses under HF treatment might be of benefit in AML treatment.

Apoptosis can be initiated by the mitochondrial (intrinsic) pathway and/or the death-receptor (extrinsic) pathway [20], [32] **.** In the intrinsic apoptosis pathway, proteins of Bcl-2 family, by changing outer and inner membrane permeability of mitochondria, regulate cytochrome C release from mitochondria, induce caspase-9/-3 activation and PARP cleavage [20], [32]. In the extrinsic pathway, death receptor (such as Fas antigen and TNF-R) triggering leads to strong caspase-8 activation, which bypasses mitochondria, leading directly to activation of caspase-3 followed by apoptosis [20], [32]. We show that HF neither affected Fas levels nor the amounts of secreted TNF-α (20–90 pg×ml^−1^/10^6^ cells/24 h) in U937 cells (F Merhi et al., unpublished results) supporting that HF does not induce AML cell apoptosis through death receptor triggering. However, small amounts of activated caspase-8, like stress-induced signals inside the cell, can degrade Bid, a member of Bcl-2 family, leading to a truncated Bid (tBid) that stimulates efficient oligomerization of Bax and activates the intrinsic pathway [24]. Bid can be cleaved not only by caspase-8 but also by other caspases, granzyme B, calpains and cathepsins [33]. Full-length Bid can also translocate to and activate the mitochondria without cleavage [24]. Here, we provide evidence that HF induced mitochondrial membrane perturbation, caspase-9/-8/-3 activation, and decreased Bid levels without the appearance of tBid in U937 cells. The results of caspase inhibition studies argue strongly that caspase-8, in contrast to caspase-9 and caspase-3, did not play a primary role in the apoptotic response to HF. Similarly, flavopiridol favors apoptosis in U937 cells through the mitochondrial pathway and independently of activation of pro-caspase-8 and Bid cleavage [34]. Together our data indicate that HF triggers cell death by apoptosis at least through the intrinsic pathway and is not dependent upon the extrinsic, procaspase-8-associated cascade. In addition, Bid has been shown to upregulate cell proliferation and to participate in cell cycle arrest arrest [24]. The downregulation of Bid protein by HF in U937 cells may therefore represent a possible mechanism by which Bid could modulate growth arrest.

Members of Bcl-2 family such as pro-apoptotic Bad, Bax, Noxa and anti-apoptotic Bcl-2, Mcl-1 proteins are important regulators of the intrinsic pathway [20]. Overexpression of Bcl-2 protein rescues U937 cells from apoptosis [35]. While Ser^136^-phosphorylation of Bad protects AML cells from apoptosis [27], dephosphorylated Bad favors apoptosis by sequestering Bcl-2 in the cytoplasm, thus preventing its binding to Bax which promotes cytochrome C release [36]. Mcl-1 also blocks cytochrome C release from mitochondria by preventing Bax activation [36]. In addition, Noxa plays an important role in cell death decision by targeting Mcl-1 for proteasomal degradation [37]. Noxa up-regulation is associated with apoptosis of LLC cells induced by HF [15]. In the present study, HF induced Ser^136^-dephosphorylation of Bad, downregulated levels of Bcl-2 and increased levels of Noxa in U937 cells. We propose that Bad and Noxa by neutralizing respectively the anti-apoptotic proteins Bcl-2 and Mcl-1 could contribute to the apoptotic effect of HF.

The cAMP-responsive element binding protein (CREB) activates the transcription of the Bcl-2 gene [38]. Phosphorylation of CREB at Ser^133^ by the serine/threonine kinase Akt1 is required for CREB-mediated transcription [39]. In addition, the Bcl-2 mRNA contains multiple predicted miRNA binding sites implicated in the inhibition of Bcl-2 expression [40]. With regard to Noxa, several regulators and control mechanisms have been described [37]. The transcription factors implicated in regulating Noxa expression include p53, hypoxia-inducible factor Hif-1α and E2F1 [37]. Upregulation of Noxa mRNA and protein is independent of p53 in various cell types [37], [41], [42]. In T cells, Noxa stimulation interferes with PKC signaling and the common γ-chain of the receptors for IL-7 and IL-15 [37]. Whether Noxa and Bcl-2 are regulated by HF at the transcriptional or post-transcriptional levels remains to be determined.

The Akt1 pathway participates in the regulation of proliferation and apoptosis [43]. Full activation of Akt1 is a multiprocess, and the final step is to phosphorylate Akt1 on two sites, Thr^308^ and Ser^473^ residues [40]. The kinases PDK2 and mTORC2 as well as Akt1 itself phosphorylate Akt1 at Ser^473^ [40]. Primary AML cells and AML cell lines express Akt1 and mTORC2 [25], [44], [45]. Activated Akt signaling protects AML cells from apoptosis [25]. Bad is a direct downstream target of Akt1 and may reflect the state of Akt signaling [25]. Akt1 negatively regulates apoptosis of AML cells through phosphorylation of Bad on Ser^136^ [27] while dephosphorylated Bad favors apoptosis [36]. As mentioned above, we found that HF downregulated the levels of Ser^136^-phosphorylated Bad in U937 cells. We thus evaluated whether HF interfered with Akt1 activity. The potential effect of HF on Akt1 has not been reported so far. We present new evidence that HF downregulates the level of the active form of Akt1 through its dephosphorylation in U937 cells. We further confirmed that HF directly inhibits Akt1 kinase activity in an ELISA assay using a recombinant Akt1 protein. Akt1 comprises a N-terminal pleckstrin homology (PH) domain, a flexible hinge between the PH and the kinase domain, a catalytic (kinase) domain and a C-terminal regulatory domain (containing a hydrophobic Ser^473^phosphorylation motif)[46], [47]. The PH domain, the hinge region and the C-terminal HM of Akt1 appear important for the inhibitory function of the specific allosteric inhibitor Akt1/2 inhibitor Akt-I-VIII [46], [48]. Further studies are required to identify the potential interactions between HF and these domains. This study therefore support a novel function of HF as a negative regulator of Akt1. Furthermore, inhibition of the Akt pathway by Akt-I-VIII induced apoptosis in U937 cells, and combined treatment with Akt-I-VIII and HF significantly increased the levels of apoptosis in comparison with treatment with HF alone. These observations strongly suggest that HF-mediated apoptosis involves inhibition of the Akt pathway. Of note, flavopiridol inhibits the levels of Ser^473^-phosphorylated Akt1 without marked inactivation of Akt1 kinase activity. In accordance, flavopiridol was previously shown to diminish phosphorylation of Akt in myeloid K562 and HL-60 cells [28], [29]. Initially described as a potent inhibitor of cyclin-dependent kinases (CDKs) by binding to the ATP-binding domain of CDKs, flavopiridol lacks absolute specificity, insofar it can inhibit other kinases, including Akt, p38MAPK, JNK, PKC and IκBα kinase [29], [49], [50]. mTOR inhibitors prevent CDK activation [51]. Thus, whether flavopiridol could inhibit mTORC2 implicated in Akt1 phosphorylation cannot be excluded. Moreover, the regulation of Akt depends on the balance between phosphorylation by kinases for activation and dephosphorylation by phosphatases for inactivation [52], [53]. Primary AML cells express various levels of protein Ser/Thr phosphatases (PP1, PP2A, PP2B, PP2C) [54]. PP2A activation by PP2A activators such as forskolin or FTY720 blocks proliferation, induces caspase-dependent apoptosis and decreases phosphorylation of Akt in AML cells [55]. Whether flavopiridol plays a role in the activation of PPs will require further investigation.

Other phosphorylated downstream targets of Akt include p53 and IκB kinase [25]. The p53 tumor-suppressor has a central role in responses to DNA damage and determination of whether cells undergo cell cycle arrest or apoptosis [56]. NB4 and OCI cell lines express p53 [57], [58] while HL-60 and U937 cells are p53 null [59]. In this study, all the cell lines were equally sensitive to the inhibitory activity of HF thus indicating that HF-mediated apoptosis operates through a p53-independent mechanism in AML cells. Akt1 can promote cell survival via activation of NF-κB, by phosphorylating IκB kinase IKKα which in turn phosphorylates and triggers Iκ-Bα degradation *via* the ubiquitin-proteasome pathway. Liberated NF-κB dimer translocates to the nucleus and activates numerous target genes involved in the control of cellular responses including apoptosis [30], [39]. Increased IκB kinase activity is associated with activated NF-κB in AML blasts thus favoring neoplastic cell survival [60]. Here, blocking NF-κB activity in NB4 cells transfected with a dominant-negative mutant form of Iκ-Bα that cannot be phosphorylated and degraded, did not prevent the inhibitory effect of HF on cell growth and survival. These data indicate that the mechanism by which HF induces apoptosis of AML cells does not involve the inhibition of NF-κB.

**Figure 9:**
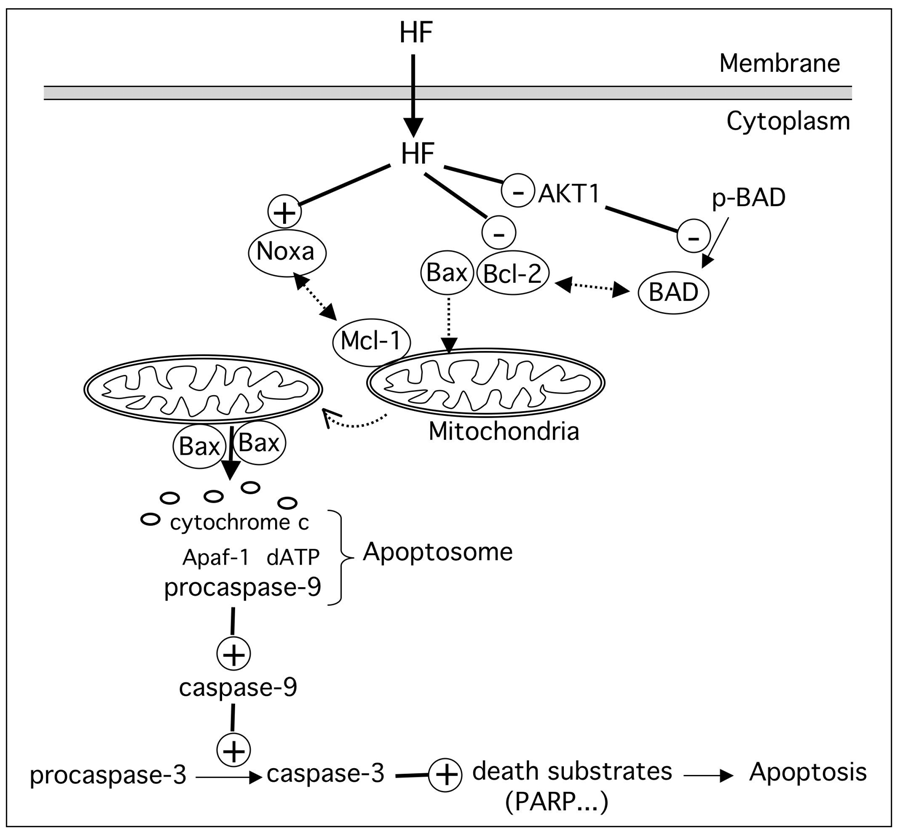
Putative model for the convergent roles of Akt1 and mitochondrial caspase-dependent signaling pathways in the induction of apoptosis by HF in AML cells. HF inactivates Akt1 leading to dephosphorylation of proapoptotic Bad, and downregulates anti-apoptotic Bcl-2 protein. Dephosphorylated Bad sequesters Bcl-2 in the cytoplasm, favoring Bax translocation to the mitochondria. HF induces upregulation of Noxa which exerts its pro-apoptotic function by neutralizing the prosurvival Mcl-1, facilitating activation of Bax. Subsequent oligomerization of Bax inserts into the outer mitochondrial membrane, and triggers cytochrome c release. Cytochrome c forms a complex with procaspase-9, Apaf-1 and dATP (apoptosome). The apoptosome activates caspase-9 which then activates caspase-3 ultimating leading to apoptosis.

In conclusion, this study emphasizes the role of HF in the induction of apoptosis in AML cells associated with inhibition of Akt1 and activation of the mitochondrial caspase-dependent cascade, and a model is proposed in Figure 9. Accordingly, recent studies have documented the convergence of protein kinases and caspases in the regulation of cell proliferation and survival [56].

Pharmacokinetics studies performed in humans have demonstrated oral bioavailability of HF [61]–[63]. Outcomes for patients with AML have not significantly improved in the past 20 years and conventional cytarabine- and anthracycline-based chemotherapy remains the gold standard. Despite the activity of these agents, 20% of patients ≤60 years and 50% of older patients fail to achieve remission with these standard agents, and only a small proportion patients have a prolonged disease-free survival [64]. Chemoresistance to standard agents appears to be related, in part, to overexpression of the protein P-gp which is the product of the MDR1 (or ABCB1) gene and the prototype of the family of ABC transporters that are involved in the phenomenon of multidrug resistance (MDR). P-gp functions by pumping certain drugs out cells, through an active energy-dependent mechanism [65], [66]. Our laboratory previously showed that HF is capable of inhibiting the functional activity of Pgp in leukemic cells [67]. Therefore, our findings may provide a new experimental basis for AML therapy that have to be confirmed with an *in vivo* leukemia models in mice. Several mouse models of human AML have been generated using transgenic, gene targeting and retroviral transduction/transplantation approaches [68]–[70]. In the model system generated by Kharas and colleagues using a myristoylated Akt1, recipients develop myeloproliferative disease, T-cell lymphoma or AML [69]. This model with enhanced Akt activation could be exploited to study the impact of HF.

Finally, dysregulation of Akt is observed in many cancers and other diseases such as diabetes, cardiovascular and neurological diseases [47]. Akt is considered as an attractive therapeutic target for cancer treatment, and novel Akt inhibitors are being developed [71], [72]. The discovery of HF as a novel Akt1 inhibitor may have implications for disease biology and treatment.

## Materials and Methods

### Ethics statement

The study was conducted and monitored in compliance with the Declaration of Helsinki 2002. Ethics approval was given by the Institutional Review Board of Paris-Saint-Antoine Hospital and of the French National Institute of Cancer (“Tumorothèque Hématologie” Paris-Saint-Antoine Hospital COHO0203 INCA 2007). Peripheral blood samples from patients were obtained after written informed consent (formulary EORTC study N°06012). Control blood samples from healthy and fully anonymized donors were purchased from the Etablissement Français du Sang (Paris-Hôtel-Dieu Hospital) and required no special written informed consent.

### Cells and treatments

Blood samples from healthy donors were obtained from the Paris-Hôtel-Dieu Hospital. Leukemic blood samples from 22 treatment-naive AML patients (12 men and 10 women; age range: 25–80) were obtained the “Tumorothèque Hématologie” Paris-Saint-Antoine Hospital. Diagnosis was established according to standard clinical criteria and the FAB committee's cytological criteria. Peripheral blood mononuclear cells (PBMCs) were separated by Ficoll-Hypaque density gradient (1.077 g/ml) centrifugation. Cells (10^6^/ml) were cultured in complete RPMI 1640 medium supplemented with 10% FCS**.**

The mycoplasma-free AML cell line U937 (ATCC CRL-1593.2; French-American-British/FAB phenotype M5 monoblast), OCI-AML3 (M4, myelomonocyte)[73], NB4 (M3, promyelocyte)[74] and HL-60 (ATCC 240-CCL; M2, myeloblast) were cultured in RPMI 1640 medium (Gibco, Paisley, UK) supplemented with 5% heat-inactivated fetal calf serum (FCS, Gibco; LPS levels <0.1 ng/ml), 2 mM L-glutamine, 1 mM sodium pyruvate and 40 µg/ml gentamycin (Gibco) in a 5% CO_2_ humidified atmosphere at 37°C. NB4 cells transfected with the Migr-eGFP vector (NB4/GFP cells) or with the Iκ-Bα (A32/36)-encoding Migr-eGFP (NB4/GFP-MAD) were generated as previously described [31]. Cells were used at passage 12 or less and harvested in log-phase growth for every experiment. Cells (1-3×10^5^/ml) were treated with various concentrations (0.1–3 µg/ml) of HF for various periods of time. Flavopirirol (100 nM) was used as a positive control for apoptosis induction. In negative control experiments, cells were treated with the same volume of EtOH (vehicle) used to dissolve HF. Caspase inhibitors were added at the beginning of the cultures and incubated for 60 min prior to the addition of HF.

### Reagents

HF (Figure 1) was purified according to the method described [75]. Stock solutions (1 mg/ml) were made in ethanol (EtOH). Flavopiridol (10 mM stock solution prepared in DMSO) was gifted by Aventis Pharmaceuticals (Bridgewater, NJ, USA). Fluorescein isothiocyanate (FITC)-conjugated anti-CD15 (80H5, mIgM), FITC-anti-CD11b (BEAR 1, mIgG1), FITC-CD44 (mIgG1, J-173), phycoerythrin (PE)-conjugated anti-CD13 (mIgG1, SJ1D1) and FITC-mIgM were obtained from Beckman-Coulter (Luminy, France). FITC-mIgG1, PE-mIgG1, anti-phospho-Ser^136^-Bad (Ser^136^, rabbit IgG), anti-PARP-1 (F-2, mIgG2a) and anti-Bad (H-168, rabbit IgG) were from Santa-Cruz (Tebu-Bio SA, Le Perray en Yvelines, France). Z-IETD-fmk (a caspase-8 inhibitor) and caspase-3/-8/-9 kit assays were obtained from R&D (Abingdon, UK). Anti-phospho-Ser^473^-Akt1 (193H12, rabbit IgG), anti-Akt1 (H-136, rabbit IgG), anti-Bid (FL-195, rabbit IgG), anti-Bcl2 (100, mIgG1) and anti-Mcl-1 (S-19, rabbit IgG) were from Cell Signaling (Hertfordshire, UK). Anti-actin (C4, mIgG1) was from ICN Biomedicals (Ohio, USA). Anti-Bax (mIgG1) was from Zymed Laboratories (San Francisco, USA). Anti-Noxa (6D619, mIgG1) was from US Biologicals/Euromedex (Mundolsheim, France) Secondary antibodies were horseradish peroxidase-conjugated from GE Healthcare Life Sciences (Picataway, NJ, USA). Z-DEVD-fmk (a caspase-3 inhibitor), Z-VAD-fmk (a general caspase inhibitor) and Akt inhibitor VIII (an Akt1/Akt2 inhibitor) were from Calbiochem (Darmsdat, Germany). Ac-LEHD-CHO (a caspase-9 inhibitor) was from AG Scientific, Inc. (San Diego, CA, USA). Active human recombinant Akt1 was supplied by Cell Sciences (Canton, MA).

### Determination of cell growth, cell death and cell cycle

Cell growth was evaluated by counting the number of viable cells (with diameters ranging from 9 to 14 µm) and dead cells (diameters ranging from 4 to 9 µm) in a Coulter Multisizer (Beckman-Coulter, Les Ullis, France). Cell cycle status was determined as described in [76] and measured with a flow cytometer (Beckman-Coulter, Luminy, France). The amount of cells in sub-G1, G0/G1, S and G2/M phases was calculated using the Win cycle software (Beckman-Coulter).

### Flow cytometry

Apoptosis was measured using the annexin-V-FITC/propidium iodide (PI) apoptosis detection kit (Beckman-Coulter). Stained cells (40,000) (AML cell lines and primary blasts) were analyzed using the flow cytometer (Beckman-Coulter). Induction of maturation was assayed by immunostaining intact HL-60, NB4 and U937cells with mAbs directed against CD11b (macrophagic marker)[77], CD13 (macrophagic marker), CD15 (granulocytic marker) and CD44 (macrophagic marker)[74], [78]. Cells to be stained (2×10^5^) were centrifuged to form a pellet and incubated with the appropriate mAbs or isotypes under saturating concentrations (1 µg) for 30 min on ice, then washed twice with PBS supplemented with 1% bovine serum albumin and 0.1% sodium azide, and fixed with 0.4% formaldehyde in PBS. Cell fluorescent intensity (40,000 cells) was measured using the flow cytometer (Beckman-Coulter). Values were given as percentages of positive cells [79].

### DNA fragmentation

Cells were washed twice with PBS and lysed in M-PER buffer (ThermoFisher Scientific, Ilkirch, France) for 60 min on ice. Lysates containing fragmented DNA were cleared by centrifugation at 10,000 g for 15 min. Supernatant samples were treated with proteinase K (500 µg/ml) at 50°C for 2 h. Thereafter, RNAse A (500 µg/ml) was added and the samples were incubated at 50°C for 90 min. Electrophoresis was performed in 1.8% agarose gels containing ethidium bromide and the bands were analyzed in an Appligen Oncor densitometer (Illkirch, France).

### Assessment of mitochondrial membrane permeability

The loss of mitochondrial membrane potential (MMP) was analyzed using the mitochondrial detection kit (Biomol GmbH, Hamburg, Germany) as described previously [14]. Following drug treatment, cells were labeled with the lipophilic fluorochrome dye JC-1. The sample's fluorescence was recorded in a Wallac Victor 2 multitask plate reader (Perkin Elmer, Norwalk, MT, USA). The depolarization of MMP is characterized by a shift from red fluorescence (FL2) to green fluorescence (FL1), i.e. a reduction in the red/green fluorescence ratio. The simultaneous detection of FL1 and FL2 was also performed by flow cytometry.

### Caspase assays

Caspase-3, -8 and -9 activities were assayed with specific substrates for caspase-3 (DEVD-pNA), caspase-8 (IETD-pNA) and caspase-9 (LEHD-pNA) in cell lysates (100 µg/assay) using the caspase cellular activity assay kits (R&D Systems) according to the manufacturer's instructions. Formation of pNA was monitored at 405 nm. Comparison of the absorbance of pNA from a treated sample with control sample allows determination of the relative increase in caspase activity.

### Western blot analysis

Cells were lysed in M-PER buffer (Pierce Biotechnology, Rockford, IL, USA) supplemented with protease and phosphatase inhibitor cocktails (Sigma). Total cell extracts were separated on 12% SDS-PAGE, transferred to nitrocellulose and blotted as described previously [80]. Immunoblotting was performed with primary antibodies diluted according to the manufacturer's instructions and samples were then incubated with HRP-coupled secondary antibodies. Blots were visualized by enhanced chemiluminescence (ECL, GE Healthcare Europe, Saclay, France) and NIH Image 1.63 software was used to quantify the intensity of the bands.

### Akt1 kinase activity assay

Akt1 kinase activity assay was carried out using the commercial kit (EKS-400A) from Enzolifesciences France (Villeurbanne, France) following the manufacturer's protocol. The assay is based on a solid phase ELISA that utilizes a specific synthetic peptide as a substrate for active human recombinant Akt1 and a polyclonal antibody that recognizes the phosphorylated form of the substrate. Controls included vehicles EtOH (for HF) and DMSO (for Akt 1/2 inhibitor/Akt-I-VIII).

### Data analysis

Data are presented as means ± SD from n independent experiments. Results are expressed as the mean ± SD. A two-tailed, paired Student's *t*-test was used to compare test and control groups. The threshold for statistical significance was set to *P*<0.05.
